# Polishing Fillings

**Published:** 1900-04-15

**Authors:** 


Polishing Fillings.—Keep a cake of calcined magnesia in the cabinet, and when the last disk of fine cuttle fish is to be used, touch it to the magnesia and you will give the gold a brilliant polish.—*Dental Hints.*

